# Chemical composition and in vitro antibacterial activity of *Pistacia terebinthus* essential oils derived from wild populations in Kosovo

**DOI:** 10.1186/s12906-016-1135-8

**Published:** 2016-05-26

**Authors:** Bledar Pulaj, Behxhet Mustafa, Kate Nelson, Cassandra L. Quave, Avni Hajdari

**Affiliations:** Department of Biology, Faculty of Mathematical and Natural Science, University of Prishtina “Hasan Prishtina”, Mother Theresa St, 10000 Prishtinë, Kosovo; Institute for Biological and Environmental Researches, Faculty of Mathematical and Natural Science, University of Prishtina “Hasan Prishtina”, Mother Theresa St, 10000 Prishtinë, Kosovo; Department of Dermatology, Emory University School of Medicine, 615 Michael St, Whitehead 105-L, Atlanta, GA 30322 USA; Center for the Study of Human Health, Emory University, 550 Asbury Circle, Candler Library 107, Atlanta, GA 30322 USA

**Keywords:** Terebinth, Limonene, Essential oil, Natural variability, MRSA, *Staphylococcus aureus*

## Abstract

**Background:**

Plant material from different organs of *Pistacia terebinthus* L., (Anacardiaceae) were collected in Kosovo with aim to analyze the chemical variability of the essential oils among native populations and to test them for potential antibacterial activity against *Staphylococcus aureus*.

**Methods:**

Essential oils obtained from leaves, pedicels, fruits and galls were analyzed by GC-FID and GC/MS. Minimum inhibitory concentration (MIC) against three clinically relevant strains of *S. aureus* (NRS385, LAC and UAMS-1) were used to evaluate the antibacterial activity of essential oils.

**Results:**

In total, 33 different compounds were identified. The main constituents were α-pinene (12.58–66.29 %), D-limonene (13.95–46.29 %), β-ocimene (0.03–40.49 %), β-pinene (2.63–20.47 %), sabinene (0.00–5.61 %) and (Z)-β-ocimene (0.00–44.85 %). Antibacterial testing of the essential oils against three clinical isolates of *S. aureus* revealed that seven of the eight samples had some activity at the concentration range tested (0.04–0.512 % v/v). The gall tissues from both sites produced the highest yield of essential oil (3.24 and 6 %), and both exhibited growth inhibitory activity against *S. aureus*. The most bioactive essential oils, which exhibited MIC_90_ values ranging from 0.032–0.128 % v/v, obtained from the fruits of the Ura e Shejtë collection site. Likewise, the leaf and pedicel essential oil from the same site was highly active with MIC_90_ values of 0.064–0.128 and 0.032–0.256 % v/v, respectively.

**Conclusions:**

Principle Component Analyses demonstrated that there is a variation in the chemical composition of essential oil depending on the plant organs from which essential oil are obtained and the geographical origin of the plant populations. The highest variability regarding the chemical composition of essential oil was found between oils obtained from different organs originating from the Prizren site. The MIC_90_ activity of *Pistacia terebinthus* was on par or superior compared with Tea Tree Oil control (0.128 % v/v), suggesting that essential oils from this species may have some potential for development as an antibacterial agent for *S. aureus* infections.

## Background

*Pistacia terebinthus* L., commonly known as terebinth or turpentine tree, is a perennial flowering plant in the Anacardiaceae family. It is a dioecious plant or shrub, growing to heights of 5 to 10 m. It grows in dry places, open woods and rocky, usually calcareous slopes and is native to the Mediterranean region and the Canary Islands [1]. Its presence has also been documented in Northern Africa, the Arabian Peninsula, and Western Asia [2]. *Pistacia terebinthus* also grows in a few habitats in southern Kosovo along the valley of the Drini river in the Prizren region. These populations are distributed in that region due to the influence of the Mediterranean climate, which comes from the Adriatic Sea through the Drini river valley, while the rest of Kosovo is characterized by a continental climate.

*Pistacia terebinthus* produces a rich mixture of substances, including resin, essential oils, proteins, organic acids, sugars, flavonoids and tannins [3–5]. The leaves are often attacked by gallicolous aphids (*Paracletus cimiciformis* von Heyden, *Forda marginata* Koch, C.L. and *Forda formicaria* von Heyden) [3], which stimulate the plant to form galls in the leaves. The galls contain a mixture of 60 % resin, 36 % tannins and 4 % essential oil [6]. Several studies have been undertaken in different locations where the species grows in the wild in order to evaluate and compare its essential oil composition [7–18]. These prior works have demonstrated that the essential oils vary in chemical composition between both plant organs and plant populations, the wild populations of *Pistacia terebinthus* in Kosovo have not yet been examined.

*Pistacia terebinthus* is known as a source of turpentine and is also used as a traditional medicine in different countries. For example, in Iran, the smoke is used as a disinfectant and air purifier [19], the leaves for the treatment of burns and the branch resin as an antiseptic for bronchitis and other respiratory and urological afflictions, as well as for anti-inflammatory and antipyretic properties [20]. The mature fruits have been used as a diuretic and for urinary inflammations, stomachache [21], stomach ulcers [22], prostate troubles, antiseptic, as a hypotensive and for headache [23]. The fruits are also consumed as a coffee substitute and the oil extracted from its fruits is used as cooking oil as well as in soap production in a certain section of Turkey [20]. There are a number of other uses of this species, including as a bioherbicide [24] and antifungal [25]. While the anti-inflammatory [26, 27] and anti-fungal [28] properties of this species have been assessed, the antibacterial effects against the human pathogen *Staphylococcus aureus* [28] has never been evaluated. The aim of this study was to analyze the chemical variability of the essential oils among native populations in Kosovo and to test them for potential antibacterial activity against *S. aureus.*

## Methods

### Plant material

The fruit pedicel, leaves, fruits and galls of *Pistacia terebinthus* were collected from August to September during the years 2013, and 2014 and in two different wild populations in Kosovo in Prizren (Prizren Fortress locality coordinates - (42°12′39″N, 20°44′42″E, elevation 474 m.a.s.l.), and Gjakovë (Ura e Shenjtë) - (42°21′18″N, 20°32′43″ E, elevation 330 m.a.s.l.). The plants were identified by B. Mustafa. Voucher specimens from two populations were deposited at the Herbarium of the Department of Biology, University of Prishtina and the Emory University Herbarium (GEO accession numbers 20,139 and 20,208).

Plant materials were air-dried in the shade at room temperature and then extracted separately using hydro-distillation method for 3 h in Clavenger type apparatus. Essential oils were stored in the dark vials and stored frozen at −18 °C, until analyzed.

### GC and GC/MS analyses

GC/FID analyses were made using an Agilent 7890A GC System equipped with an FID detector (Agilent Technologies). The separation was conducted on a HP-5MS column 30 m × 0.25 mm with 0.25 mm film thickness. Helium was used as the carrier gas with an initial flow rate of 0.6 mL/min and subsequently at a constant pressure of 16.6 psi. The front inlet was maintained at 250 °C in a split ratio of 50:1. The GC oven temperature increased from 60 °C to 260 °C at a rate of 5 °C/min, and the FID operated at 250 °C with an air flow of 350 mL/min and a hydrogen flow of 35 mL/min. The injection volume was 1.0 μL. GC/MS analyses were performed using an Agilent 7890A GC system coupled to a 5975C MSD (Agilent Technologies). The ionization energy was 70 eV with a mass range of 40–400 m/z. The separation was conducted with the same column and temperature program as for the analytical GC.

Identification of each component of the essential oil was made by comparing their Kovats retention indexes with those in literature [29]. Calculation of the Kovats index was made based on a linear interpolation of the retention time of the homologous series of n-alkanes (C9 - C28) under the same operating conditions. The components were also identified by comparing the mass spectra of each constituent with those stored in the MS library search (NIST 08.L and WILEY MS 9th) and with mass spectra from the literature [29].

### Principle component analysis

Principle Component Analysis (PCA) was used to evaluate the chemical composition variability between the essential oils obtained from different plant organs and geographical origins. Essential oil constituents present at concentrations higher than 1 % of total essential oil were subjected to PCA using the statistical analysis software XLSTAT version 2015.2.02.

### Microbiological activity

#### Strains and growth conditions

Three strains of *Staphylococcus aureus* (NRS385, LAC and UAMS-1) were used in this study. NRS385 is a virulent USA500 healthcare associated methicillin-resistant *S. aureus* (HA-MRSA) isolate. LAC is a virulent USA300 community associated MRSA (CA-MRSA) isolate. UAMS-1 is a heavy biofilm producing clinical methicillin sensitive (MSSA) osteomyelitis isolate from the USA100 lineage. Together, these three strains represent different phenotypes of *S. aureus* and serve as useful indicators for anti-staphylococcal activity in an initial screen.

Strains were grown from freezer stock onto tryptic soy agar (TSA) plates and then overnight cultures were grown in tryptic soy broth. Antibacterial testing (described below) was conducted in cation-adjusted Mueller Hinton broth (CAMHB). All cultures were grown at 37 °C.

#### Determination of minimum inhibitory concentration (MIC)

*Pistacia terebinthus* essential oils were tested for any growth inhibitory effects by measuring their minimum inhibitory concentration (MIC) against three clinically relevant strains of *S. aureus.* The Clinical Laboratory Standards Institute (CLSI) M100-S23 guidelines for microtiter broth dilution testing were followed, with the adjustment that activity of the essential oils is reported as % v/v (percent volume essential oil/volume of the test well) and antibiotic controls reported as μg/mL [30]. Controls included the vehicle (a ratio of 1:200 Tween 80 to DMSO), Tea Tree Oil (Thursday Plantation, Australia) and antibiotics: Vancomycin (Van) and Kanamycin (Kan) (MP Biomedical). The essential oil was mixed with vehicle solution at the ratio of 1:10 essential oil to vehicle to enhance miscibility of the essential oil with the growth media.

Overnight cultures in CAMHB were diluted in fresh CAMHB and standardized by OD to 5 × 10^5^ CFU/mL, and this was confirmed by plate counts. Two-fold serial dilutions were performed on a 96-well plate to achieve a test range of 0.04–0.512 % v/v for essential oils and 0.05–64 μg/mL for antibiotics. Plates were incubated at 37 °C for 18 h. Plates were read at an optical density (OD) of 600 nm in a Cytation 3 multimode plate reader (Biotek) at 0 and 18 h post inoculation.

The following formula, which takes into account the impact of test material color on the OD, was used as previously described [31] with OD_t18_ = OD of the test well at 18 h, OD_t0_ = OD of the test well at 0 h, OD_vc18_ = OD of the vehicle control well at 18 h, and OD_vc0_ = OD of the vehicle control well at 0 h.

$$ \%\;\mathrm{Inhibition}=\left[1-\left(\frac{ODt18-ODt0}{ODvc18- ODvc0}\right)\right]\times 100 $$

MIC_50_ and MIC_90_ values were assigned based on the concentration at which at least 50 or 90 % inhibition of growth was observed as determined by OD, respectively. All tests were performed in triplicate, and on two separate occasions. Results are reported by concentration at MIC_50_ or MIC_90_ levels, representing the concentration necessary for 50 or 90 % inhibition of growth, respectively.

## Results

### Chemical composition

The chemical composition of essential oils from different organs of *Pistacia terebinthus* is reported in Table 1. In total, 33 volatile components were identified and are listed based on their Kovats Indices (KI). The number of identified components varies and depends both on the plant organ and population origin. The plant population from the Ura e Shejtë site yielded a smaller number of identifiable compounds in the essential oil than that of the Prizren population.

**Table 1:** Composition (%) of the essential oils from leaves, pedicels, fruits and galls of *P. terebinthus* from two locations

			Prizren				Ura e Shejtë			
	KI^a^	Compounds^b^	Pedicels	Fruits	Leaves	Galls	Pedicels	Fruits	Leaves	Galls
1	926	Tricyclene	0.85	0.33	0.00	0.00	0.16	0.07	0.00	0.10
2	939	α-Pinene	45.36	21.43	47.28	52.48	22.80	12.58	32.84	66.29
3	954	Camphene	3.04	1.38	1.66	0.00	0.59	0.34	0.42	0.48
4	975	Sabinene	5.61	1.56	1.51	0.00	1.16	0.35	0.46	0.22
5	979	β-Pinene	20.47	6.86	12.46	0.00	11.24	2.63	3.09	11.11
6	990	β-Myrecene	2.83	2.83	2.92	0.15	0.86	1.93	0.60	2.72
7	1002	α-Phellandrene	0.15	0.15	0.00	1.25	0.00	0.12	0.00	0.00
8	1029	Limonene	13.95	15.65	16.69	22.60	46.29	23.87	29.02	17.32
9	1037	(Z)-β-Ocimene	0.13	2.23	0.26	0.00	13.84	44.85	29.25	0.04
10	1044	(E)-β-Ocimene	0.28	40.49	3.22	0.75	0.45	9.59	3.22	0.03
11	1059	γ-Terpinene	0.25	0.31	0.00	4.18	0.00	0.00	0.00	0.00
12	1088	Terpinolene	0.25	0.48	0.25	5.73	0.05	0.15	0.00	0.58
13	1096	Linalool	0.25	0.23	0.00	0.00	0.00	0.14	0.00	0.00
14	1132	allo-Ocimene	0.54	0.29	0.00	0.77	0.00	0.13	0.00	0.00
15	1142	(E)-Epoxy-ocimene	0.14	0.13	0.00	0.00	0.05	0.07	0.00	0.06
16	1146	Camphor	0.52	0.66	0.00	0.32	0.11	0.04	0.00	0.05
17	1169	Borneol	0.48	0.48	0.24	0.13	0.18	0.28	0.00	0.09
18	1179	Terpineol-4	0.00	0.26	0.00	0.99	0.10	0.78	0.00	0.60
19	1186	α–Terpineol	0.00	0.10	0.00	1.34	0.00	0.00	0.00	0.00
20	1205	Verbenone	0.00	0.08	0.00	1.08	0.00	0.00	0.00	0.00
21	1205	(Z)-Piperitol	0.19	0.12	0.00	0.50	0.00	0.00	0.00	0.00
22	1282	Bronyl acetate	0.30	0.19	0.00	0.70	0.00	0.00	0.00	0.00
23	1285	Isobronyl acetate	0.13	0.00	0.00	1.70	0.00	0.00	0.00	0.00
24	1387	β-Cubebene	0.83	1.37	2.57	0.23	0.00	0.00	0.00	0.00
25	1419	Cariophyllene	1.18	0.37	3.94	0.76	0.00	0.00	0.00	0.00
26	1452	α–Humulene	0.42	0.29	0.40	1.24	0.00	0.00	0.00	0.00
27	1484	Germacrene D	0.11	0.00	0.00	1.52	0.00	0.00	0.00	0.00
28	1524	δ-Cadinene	0.15	0.19	2.11	0.88	0.00	0.00	0.00	0.00
29	2051	Unknown 1	0.94	0.26	0.00	0.00	0.10	0.17	0.22	0.00
30	2104	Unknown 2	0.06	0.78	0.45	0.34	0.61	0.25	0.44	0.07
31	2300	n-Tricosane	0.20	0.20	1.60	0.33	0.11	0.15	0.00	0.00
32	2400	n-Tetracosane	0.23	0.16	1.18	0.00	1.08	0.21	0.24	0.22
33	2500	n-Pentacosane	0.16	0.16	1.25	0.00	0.22	1.31	0.20	0.03
		Yield (% v/w)	0.33 %	0.02 %	0.10 %	3.24 %	0.84 %	0.12 %	0.22 %	6 %
		Monoterpenes	93.85	94.11	86.24	87.92	97.48	96.67	98.90	98.94
		Oxygenated Monoterpenes	1.43	1.94	0.24	4.37	0.39	1.25	0.00	0.74
		Sesquiterpenes	2.69	2.22	9.03	4.63	0.00	0.00	0.00	0.00
		Other	2.03	1.74	4.49	3.08	2.13	2.08	1.10	0.32

^a^Kovats indices calculated against a C9- C28 n-alkanes mixture on the HP5 MS column

^b^Compounds are listed in order of elution from a HP-5MS column and their percentages were obtained by FID peak-area normalization

The most abundant compound was α-pinene, which ranged from 52.5 to 66.3 % in galls, 23.8–47.3 % in leaves, 22.8–45.3 % in pedicels, and 12.5–21.4 % in fruits. Limonene was the second most abundant compound found in the essential oils, with levels ranging from 13.9–46.2 % in pedicels, 15.6–23.8 % in fruits, 16.6–29.0 % in leaves and 17.3–22.6 % in galls. (Z)-β-Ocimene was present in the fruits, leaves, pedicels and galls from both locations, but the concentration differed in the same organs from each location, with the higher concentration (44.8 %) being found in fruits and 29.2 % in leaves from Ura e Shejtë, while the Prizren sample had 2.2 % in fruits and 0.2 % in leaves. (E)-β-Ocimene, on the other hand, was found in high levels in fruits (40.4 %) from the Prizren collection site, but not from the Ura e Shejtë population (9.5 %).

### Chemical variability

Principal component analysis (PCA) was used to identify the possible relationships between volatile compounds and the geographic locations of the plants. PCA demonstrates that the first two principal axes represented 54.4 % of the total variance. The first axis (30.6 % of the total variance) accounted for positive contributions of δ-cadinene, α-pinene, α-humulene, isobronyl acetate, germacrene D, verbenone, α- terpineol, γ-terpinene, α-phellandrene, terpinolene, D-Limonene, and negative contributors of (Z)-β-ocimene and β-ocimene. The second axis (23.8 % of the total variance), accounted for positive contributions of n-tricosane, cariophyllene, β-cubebene, camphene, β-pinene, β-myrecene, sabinene, and n-tetracosane, while for negative contributions of (Z)-β-ocimene and β-ocimene.

**Fig. 1:**
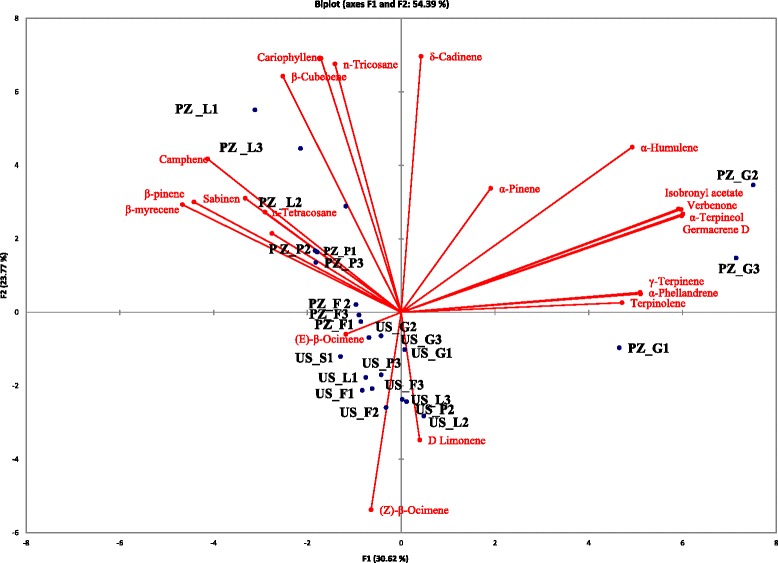
Principle component analysis of selected terpenes (higher than 1 %) isolated from the samples of *Pistacia terebinthus* L. from two populations. PZ-Prizren, US- Ura e Shejtë, L-Leaves, G-Galls, F-Fruits, P- Pedicels

The two dimensional axial system of analysis identified two groups of populations based on the chemical composition of their essential oils (Fig. 1). The samples originated from Ura e Shejtë location were quite uniform and clustered together, thus (Z)-β-Ocimene, limonene and, β-ocimene were the principal compounds that contributed to separating the Ura e Shejtë samples from those from Prizren. The highest variability regarding the chemical composition of essential oil was found between oils obtained from different organs originating from the Prizren site (dominated by δ-cadinene, α-pinene, α-humulene, caryophyllene etc.). Thus samples obtained from fruits and pedicels have quite similar chemical composition with those originating from Ura e Shejtë, while the most diverse samples were those from leaves and galls originating from Prizren (Fig. 1).

### Antibacterial activity

**Fig. 2:**
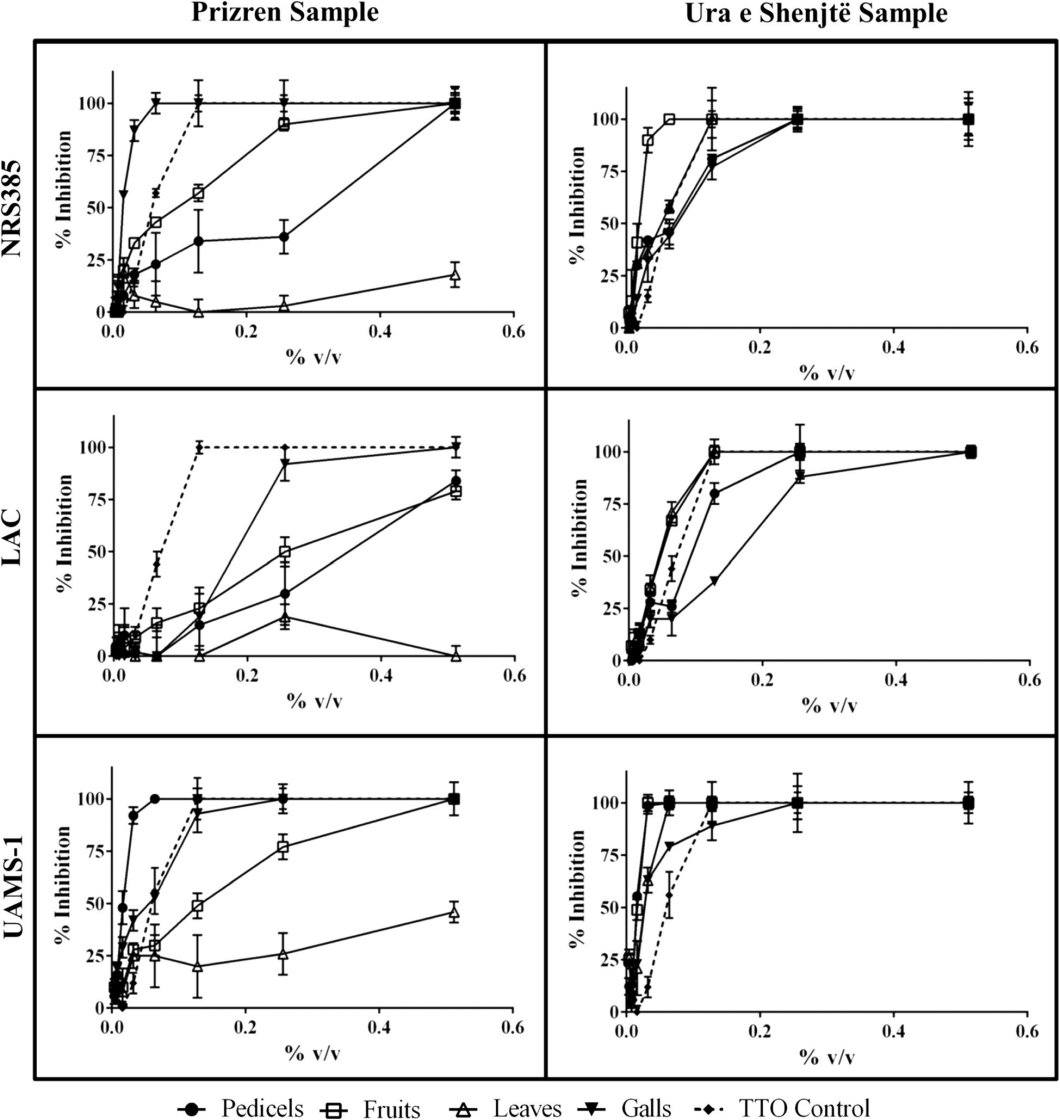
Growth inhibitory activity of *Pistacia terebinthus* essential oils from different tissues and collection sites against three clinical isolates of *Staphylococcus aureus*

Dose dependent growth inhibitory activity was observed in all of the essential oils tested, with the exception of the leaf samples from the Prizren sample site (Fig. 2). Essential oils were most active against the UAMS-1, which is a methicillin sensitive strain of *S. aureus*. The antibacterial testing results are reported as MIC_50_ and MIC_90_ values in Table 2. Overall, the essential oils of the Ura e Shejtë population were more active than those of the Prizren site, with the lowest MIC_50_ and MIC_90_ detected at 0.016 and 0.032 % v/v in the pedicel and fruit essential oils. Notably, the pedicel essential oil is much higher in limonene content for Ura e Shejtë (46.29 %) in comparison to Prizren (13.95 %). This was also the case for the fruit essential oil, with a limonene content of 23.87 % in the Ura e Shejtë sample versus 16.69 % in Prizren.

**Table 2:** Minimum inhibitory concentration of *Pistacia terebinthus* essential oils against *Staphylococcus aureus*

	Test Group	Test Agent	NRS385		LAC		UAMS-1	
			MIC_50_	MIC_90_	MIC_50_	MIC_90_	MIC_50_	MIC_90_
Essential oil (% v/v)	Prizren	Pedicels	0.512	0.512	0.512	.512	0.016	0.032
		Fruits	0.128	0.256	0.256	-	0.128	0.512
		Leaves	-	-	-	-	-	-
		Galls	0.016	0.064	0.256	0.256	0.064	0.128
	Ura e Shenjtë	Pedicels	0.128	0.256	0.128	0.256	0.016	0.032
		Fruits	0.032	0.032	0.064	0.128	0.016	0.032
		Leaves	0.064	0.128	0.064	0.128	0.032	0.064
		Galls	0.128	0.256	0.256	0.256	0.032	0.128
	Control	Tea Tree Oil	0.064	0.128	0.128	0.128	0.064	0.128
		Kanamycin	-	-	2	4	2	4
		Vancomycin	8	8	4	8	4	4

“-“: MIC not detected at the dose range tested

## Discussion

Previous studies have demonstrated that there is a variation in the chemical composition of essential oil depending on the plant organs from which essential oil are obtained and the geographical origin of the plant populations of *Pistacia terebinthus* According to Couladis et al. [9], the chemical composition of the essential oil of *Pistacia terebinthus* growing wild in Turkey differed based on the organs from which the essential oils are obtained (e.g., young shoots, flowers, unripe and ripe fruits). The main compounds included limonene (3.0, 9.4, 34.2 and 32.8 %), α-pinene (5.3, 12.4, 15.6 and 5.3 %), β-pinene (1.4, 8.0, 11.5 and 22.5 %) and germacrene D (trace, 19.9, 3.5 and 4.6 %), respectively. In Sardinia, Italy [32], the main components were α-pinene (66 %) in twigs, followed by fruiting twigs (54.8 %) and leaves (16.4 %). β–pinene was another compound with a high percentage 22.5 % in fruitful twigs 13.5 % in leaves and 5.9 % in twigs. Ulukanli et al. [33] shows that the essential oil obtained from leaves of *Pistacia terebinthus* L. spp. *palaestina* (Boisse.) is dominated by α-pinene (19.9 %), sabinene (15,4 %), terpinen*-*4-ol (9.6 %) and β*-*pinene (8.5 %). The leaf essential oil obtained from Tunisia [18] was dominated by α-pinene (19.2 %), α*-*terpinene (41.3 %), α*-*terpinolene (8.0 %) and δ*-*terpinene (4.4 %), while leaf essential oil from Turkey [34] was dominated by α-cadinol (6.9 %), followed by phytol (5.4 %), δ-cadinene (5.1 %), α-terpineol (5.0 %), and bornyl acetate (4.4 %).

The essential oil yields from the Kosovar samples also differed between plant organs and collection sites. In the organs from the Prizren population, the yield ranged from 0.02 % in fruits, 0.10 % in leaves, 0.33 % in pedicels and 3.24 % in galls, while in the other location (Ura e Shejtë), the yield was 0.12 % in fruits, 0.22 % in leaves, 0.84 % in pedicels and 6 % in galls. Previous work has shown that the yield of *Pistacia terebinthus* differs depending from which plant organs the oil were extracted and the origin of the plant population. For example, the leaf essential oil from a Tunisian population yielded 0.25 % w/w [18], while the leaves, twigs and fruitful twigs of Sardinian population gave 0.01, 0.05 and 1.5 v/w respectively [32], young shoots, flowers, unripe and ripe fruits gave 0.74, 0.70, 0.54 and 0.73 % w/w respectively [9], while the essential oil yield from *Pistacia terebinthus* L. spp. *palaestina* leaves was 0.1 % v/w [33].

Regarding the antibacterial activity detected, limonene and limonene-rich oil extracts have been implicated as effective antimicrobial agents against *Staphylococcus aureus* [35–38]. Here, we found that the antibacterial activity of the fruit essential oil was higher for the population from Ura e Shejtë (MIC_90_ of 0.032–0.128 % v/v) in comparison to Prizren (MIC_90_ of 0.256–0.512 % v/v). The main difference in essential oil composition of the two fruit samples is due to the abundance of two similar monoterpenes, with (Z)- β-ocimene being highest in the bioactive sample (44.85 % in Ura e Shejtë versus 2.23 % in Prizren) and (E)- β-ocimene being highest in the nonactive fruit essential oil sample (40.49 % in Prizren versus 9.59 % in Ura e Shejtë). The percent composition of limonene in the fruit essential oils is also slightly higher for the Ura e Shejtë population (23.87 versus 15.65 %). Most notably, two of the essential oils from the Ura e Shejtë site (leaves and fruits) performed on par with or better than our positive control of Tea Tree Oil (TTO) in all three clinical isolates of *S. aureus* tested, indicating that these oils may have some potential for further development as antibacterial agents.

Interestingly, gall essential oils obtained by the hydrodistillation gave higher yields in comparison with those obtained from other plant organs (leaves, pedicels and fruits). Gall essential oils (especially those originated from the Prizren site) demonstrated promising antimicrobial activity on par with or superior to TTO. As a key structural response site for defense against aphids, it is not surprising that a higher yield of bioactive secondary metabolites are concentrated in the gall tissue. Furthermore, the highest yield of essential oil was found in the gall tissues.

## Conclusion

This study has demonstrated that there is a variation in the chemical composition of essential oil depending on the plant organs from which essential oil are obtained and the geographical origin of the plant populations of *P. terebinthus*. The highest variability regarding the chemical composition of essential oil was found between oils obtained from different organs originating from the Prizren site.

Most notably, two of the essential oils from the Ura e Shejtë site (leaves and fruits) performed on par with or better than our positive control of Tea Tree Oil (TTO) in all three clinical isolates of *S. aureus* tested, indicating that these oils may have some potential for further development as antibacterial agents.

The antibacterial activity of *Pistacia terebinthus* essential oils against *Staphylococcus aureus* supports the use of this species in various traditional medicinal applications, especially concerning its use as an anti-inflammatory agent and antiseptic. Further research is necessary to examine the safety of its use for topical or oral applications. Importantly, future studies on the bioactivity of this species must take into account the variability in chemical composition of the essential oils both between collection sites and plant tissues as this can result in large differences in bioactivity of the test product.

## Abbreviations

%, percentage; % v/v, percent of volume/volume; °C, degree Celsius; μg/mL, microgram per milliliter; CAMHB, cation-adjusted Mueller-Hinton broth; CFU/mL, colony-forming unit per milliliter; CLSI, clinical laboratory standards institute; DMSO, dimethyl sulfoxide; eV, electron volt; GC/MS, gas chromatography coupled with mass spectrometer detector; GC, gas chromatography coupled; GC-FID, gas chromatography coupled with flame ionization detector, m.a.s.l., meter above sea level, m/z, mass-to-charge ratio; MIC, minimum inhibitory concentration; mL/min, milliliter per minute; MS, mass spectrometer detector; NIST, national institute of standards and technology; OD, optical density; PCA, principle component analysis; TSA, tryptic soy agar; μg, microgram; μl, microliter.
